# Ectopic Thyroid Tissue Mimicking a Carotid Body Paraganglioma

**DOI:** 10.7759/cureus.81740

**Published:** 2025-04-05

**Authors:** Chaymae Mezdid, Drissia Benfadil, Azzedine Lachkar, Fahd El Ayoubi

**Affiliations:** 1 Department of Otolaryngology, Head and Neck Surgery, Centre Hospitalier Universitaire (CHU) Mohammed VI, Faculty of Medicine and Pharmacy, Mohammed I University, Oujda, MAR

**Keywords:** carotid artery, ectopic thyroid gland, histology, imaging, paraganglioma

## Abstract

Ectopic thyroid tissue (ETT) in the lateral neck is a rare condition. We present the case of a 68-year-old woman who was admitted to our department with an isolated left lateral cervical swelling evolving for over eight months. CT scan and cervical MRI concluded to a paraganglioma. In surgery, a complete removal of the tumor was performed; however, it did not have the aspect of a paraganglioma. Then, histopathology revealed an ectopic thyroid adenoma.

Even though ETT is very rare, it must be mentioned in the differential diagnosis of cervical paraganglioma.

## Introduction

The thyroid gland is the first endocrine gland to develop. It appears around the third week of gestation as a thickening of the endodermal epithelial cells on the median surface of the floor of the primitive pharynx [1].

Ectopic thyroid tissue (ETT) can occur along the embryological descent of the thyroid, most commonly in the tongue (lingual thyroid), but also in the cervical region or even in rare locations such as the mediastinum or submandibular area [2]. In this article, we present the case of a patient with laterally located ETT imitating a cervical paraganglioma while having a normal thyroid gland.

To our knowledge, only five cases with a similar diagnosis have been reported in the literature to date [3].

## Case presentation

A 68-year-old woman with a past medical history of diabetes, high blood pressure, and atrial fibrillation consulted our department with an isolated left lateral cervical swelling that had been evolving for over eight months. Clinical examination revealed a firm, painless, non-pulsatile mass. Examination with rigid endoscopes was unremarkable. Blood tests were normal, including thyroid function tests and plasma free metanephrine/normetanephrine levels (Table 1).

**Table 1:** Thyroid function tests and plasma free metanephrine/normetanephrine levels TSH: Thyroid-stimulating hormone; T4: Thyroxine

Parameter	Patient value	Normal range
Plasma free metanephrine (nmol/L)	0.45	<1.07
Normetanephrine (nmol/L)	<0.10	<0.33
TSH (uIU/mL)	1.35	0.35-4.94
T4 (pg/mL)	7.90	7-14.8

**Figure 1:**
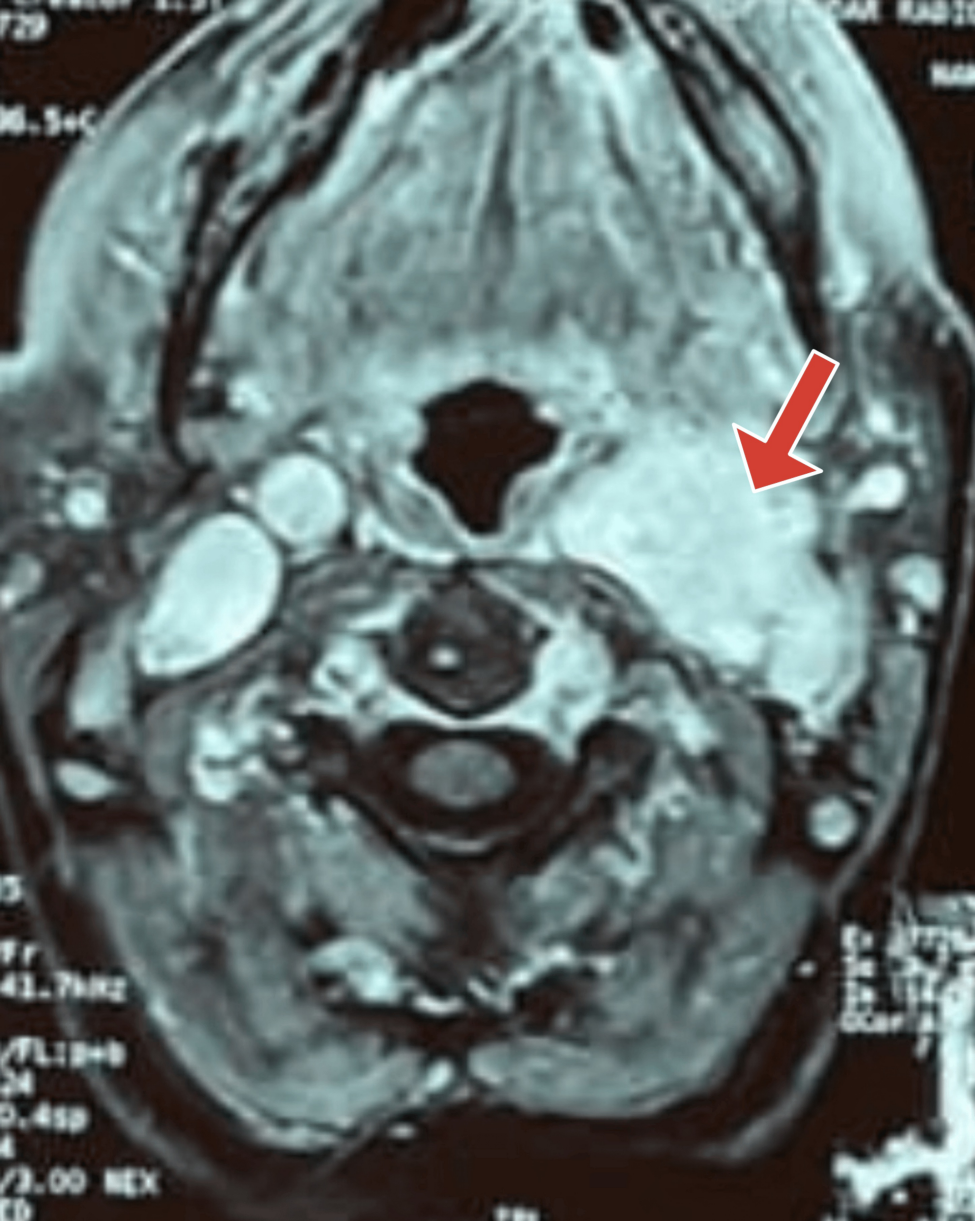
CT scan of the neck showing a soft tissue mass (red arrow) at the left carotid bifurcation splaying the ICA and ECA ICA: Internal carotid artery; ECA: External carotid artery

Her CT scan revealed a heterogenous enhancing mass at the left carotid bifurcation splaying the internal and external carotid arteries (Figure 1).

**Figure 2:**
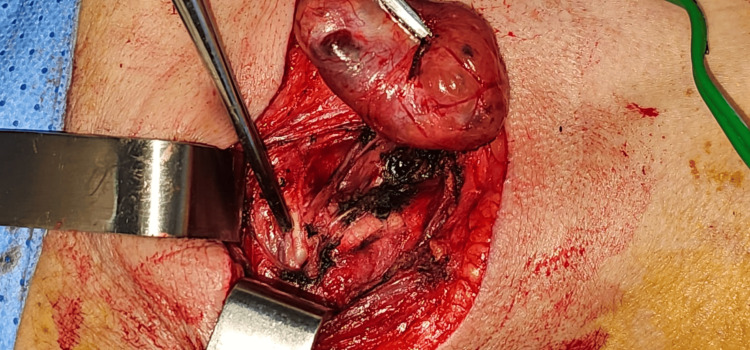
Photograph taken intraoperatively showing the successful dissection of the mass with the preservation of all the vessels and nerves

Intraoperatively, the mass was located at the carotid bifurcation and was easily dissected off the carotid vessels (internal and external carotid arteries) without any vascular injuries and with preservation of lower cranial nerves (vagus nerve, hypoglossal nerve, ansa hypoglossi) (Figure 2). 

Her postoperative course was uncomplicated, and she didn't develop any signs of hypothyroidism. She was discharged home after four days.

**Figure 3:**
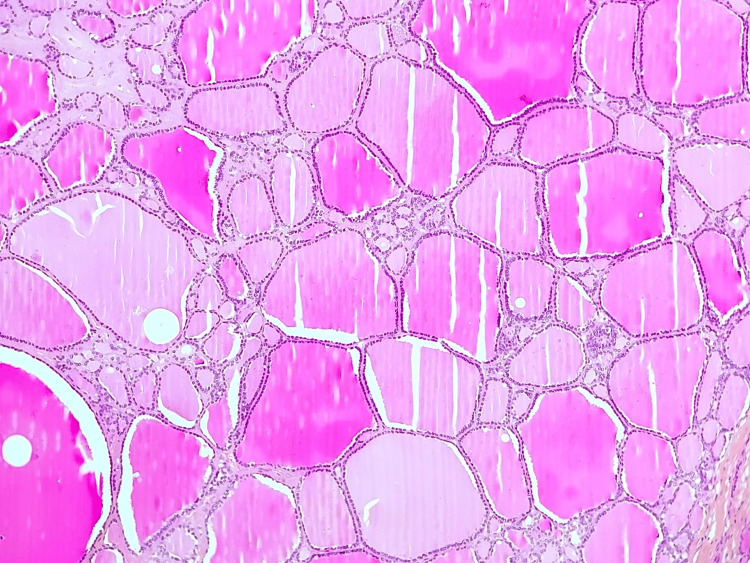
Microscopic examination of the cervical mass revealing regular thyroid tissue with multiple follicles filled with eosinophilic material. The follicular epithelium consisted of cuboidal cells with uniformly small nuclei and no atypia (H&E, x200) H&E: Hemotoxylin and eosin

Histopathological examination revealed regular thyroid tissue with multiple follicles filled with eosinophilic material. The follicular epithelium consisted of cuboidal cells with uniformly small nuclei and no atypia, with no evidence of paraganglioma/carotid body tumor (Figure 3). 

Thus, the diagnosis of ETT was retained.

## Discussion

ETT in the lateral neck is a rare condition, accounting for only about 1-3% of all ETT cases identified at cervical levels I to V [4]. Its manifestation at the carotid bifurcation is even more unusual, and our literature review revealed only five previously reported cases [2,3].

Several theories have been suggested to explain the origin of ETT, including displacement during the course of embryonal development or the detachment of thyroid tissue from the lateral lobes of the thyroid, which remains lateralized in the neck [2-5].

In most reviewed cases of ETT, the mass was located on the right side, unlike in our case, and most patients were asymptomatic, consistent with our findings.

Although diagnosing ETT can be challenging since differentiation from carotid body paraganglioma is often unclear even with an arteriograph, it is still necessary to characterize the tumor mass with an accurate preoperative diagnosis. CT and MRI are highly sensitive and specific diagnostic methods, especially when utilized together [2].

The incidence of cancer appears to be higher in ETT, with approximately 80% of these malignancies being papillary carcinoma [6].

## Conclusions

ETT should be considered in the differential diagnosis of lateral neck masses, even if it is very rare especially for the high risk of malignancy. It is also important to differentiate the ETT in the lateral neck from paraganglioma based on valuable imaging methods. Surgical excision of the lesion followed by histological examination is essential for confirming the diagnosis.
